# NSD1 in erythroid differentiation and leukemogenesis

**DOI:** 10.1080/23723556.2020.1809919

**Published:** 2020-09-14

**Authors:** Samantha Tauchmann, Marwa Almosailleakh, Juerg Schwaller

**Affiliations:** University Children’s Hospital Basel (UKBB), Department of Biomedicine, University of Basel, Basel, Switzerland

**Keywords:** Histone methyltransferase, erythroleukemia, erythroid differentiation, NSD1, GATA1

## Abstract

We have uncovered a novel role for the nuclear receptor-binding SET domain protein 1 (NSD1) in human and murine erythroid differentiation. Mechanistically, we found that the histone methyltransferase activity of NSD1 is essential for chromatin binding, protein interactions and target gene activation of the erythroid transcriptional master regulator GATA1.

The genetic code is stored inside the nucleus of cells and forms compact structures, known as chromatin, composed of a densely folded complex of DNA ribbons and small proteins called histones. DNA methylation and histone posttranslational modifications elicit heritable changes in gene expression, without actual changes in the DNA sequence, a phenomenon referred to as epigenetics. Precise deposition and timing of epigenetic modifications are essential during development and maintenance, as they have been implicated in fundamental biological processes, such as proliferation, differentiation and DNA damage responses. Addition of methyl groups to lysine residues on histone tails regulates DNA accessibility and thus, has a profound impact on the transcriptional regulation of genes. This process is conducted by multiple histone lysine methyltransferase (KMT) enzymes and results in either activation or repression of gene expression.^1^ Recurrent genetic alterations in KMT leading to aberrant histone modifications are frequently detected in acute myeloid leukemia (AML) and have been shown to act as drivers of the disease.^2^

The nuclear receptor-binding SET domain protein 1 (NSD1) has been characterized as a mono- and di-methyltransferase of histone H3K36.^3^ Germline loss of function (LOF) mutations of *NSD1* are linked to SOTOS childhood overgrowth syndrome, while acquired alterations are frequently detected in various human cancers. Reduced NSD1 activity, as a result of putative LOF mutations, is detected in head and neck cell cancers, while aberrant DNA promoter hypermethylation is associated with renal clear cell carcinoma. In hematological malignancies, the *NSD1* gene is not only a target of LOF mutations but is also involved in a recurrent t(5;11)(q35;p15) chromosomal translocation associated with aggressive pediatric AML. The resulting fusion composed of the N-terminus of the nucleopore 98 (NUP98) and the C-terminal NSD1 SET domain has leukemogenic activity.^3^

In order to decipher its role in hematopoiesis, we inactivated NSD1 in human and mouse hematopoietic cells.^4^ Reduced *NSD1* mRNA expression in human CD34^+^ hematopoietic stem and progenitor cells impaired the cells’ *in vitro* erythroid differentiation and increased their clonogenic potential. Remarkably, *Vav-iCre*-controlled inactivation of the *Nsd1* (*Nsd1^-/-^*) gene in the hematopoietic system of the mouse induced a fully penetrant erythroleukemia-like disease characterized by anemia, thrombocytopenia and multi-organ erythroblast infiltration. Interestingly, a recent study has reported that doxycycline-regulated overexpression of an *H3K36M* onco-histone transgene in mice resulted in a highly similar, fully penetrant erythroleukemia-like phenotype.^5^ Notably, the H3K36M-mutant nucleosomes dominantly inhibit the enzymatic activities of H3K36 KMTs, including NSD1, resulting in reduced H3K36 mono- and di-methylation. The striking phenotypic similarities between these two models strongly indicate that appropriately regulated H3K36 methylation is crucial for normal terminal erythroid differentiation.

To identify the underlying molecular mechanisms, we compared the impact of adding-back a wildtype *Nsd1* and a catalytically inactive *Nsd1^N1918Q^* mutant cDNA on *in vitro* erythroid differentiation. Only wildtype NSD1 restored terminal differentiation of *Nsd1*^-/-^ erythroblasts associated with significantly increased chromatin binding and activation of target genes of the erythroid master regulator GATA1. While loss of the NSD1 catalytical function severely impaired GATA1 transactivation activity and erythroid differentiation, overexpression of exogenous GATA1 was sufficient to partially overcome the differentiation block. Comparative analysis of GATA1-bound proteins in *Nsd1*^-/-^ erythroblasts upon retroviral *Nsd1* expression revealed reduced interaction with multiple transcriptional co-repressors such as the v-Ski avian sarcoma viral oncogene homolog (SKI), CBFA2/RUNX1 partner transcriptional co-repressor 3 (CBFA2T3), nuclear receptor corepressor 1/2 (NCOR1/2) and the histone deacetylase 3 (HDAC3).^4^ In addition, NSD1 restoration led to an increase in H3K36me1/2 activation marks, coupled with a decrease in H3K27me repressive marks, suggesting the formation of transcription promoting chromatin structure. These observations suggest that mono- and di-methylation of H3K36 by NSD1 is essential for proper transactivation of GATA1 controlled genes that are critical for terminal erythroid maturation.

Extensive biochemical *in vitro* experiments revealed that NSD1-mediated H3K36me2 marks are required for the recruitment of DNA methyltransferase 3 alpha (DNMT3A) and maintenance of DNA methylation.^6^ Genetic ablation of *Nsd1* and its paralogue *Nsd2* in murine cells resulted in a redistribution of DNMT3A to H3K36me3-modified gene bodies and a reduction in the methylation of intergenic regions. Notably, both blood samples of SOTOS patients and NSD1-mutant cancer cells exhibited hypomethylation of intergenic DNA. This suggests that reduced H3K36 methylation connects human cancers and developmental overgrowth through aberrant intergenic CpG methylation. Interestingly, DNA methylation by DNMT3A or the Tet methylcytosine dioxygenase (TET2) was shown to regulate hematopoietic differentiation by controlling accessible binding sites for hematopoietic transcription factors including GATA1.^7^ Moreover, very recent work has shown that precise DNA methylation patterning can control the binding and regulation of GATA1 activity.^8^ Collectively, these findings suggest that the erythroleukemia-like phenotype in *Nsd1*^-/-^ mice is probably the consequence of altered crosstalk between histone and DNA methylation. Although many pieces of the puzzle are missing, these observations suggest that NSD1 regulates erythroid differentiation by creating a multi-layered context-dependent epigenetic topography involving histone and DNA methylation, affecting the activity of master transcription factors such as GATA1 (Figure 1).

**Figure 1.**
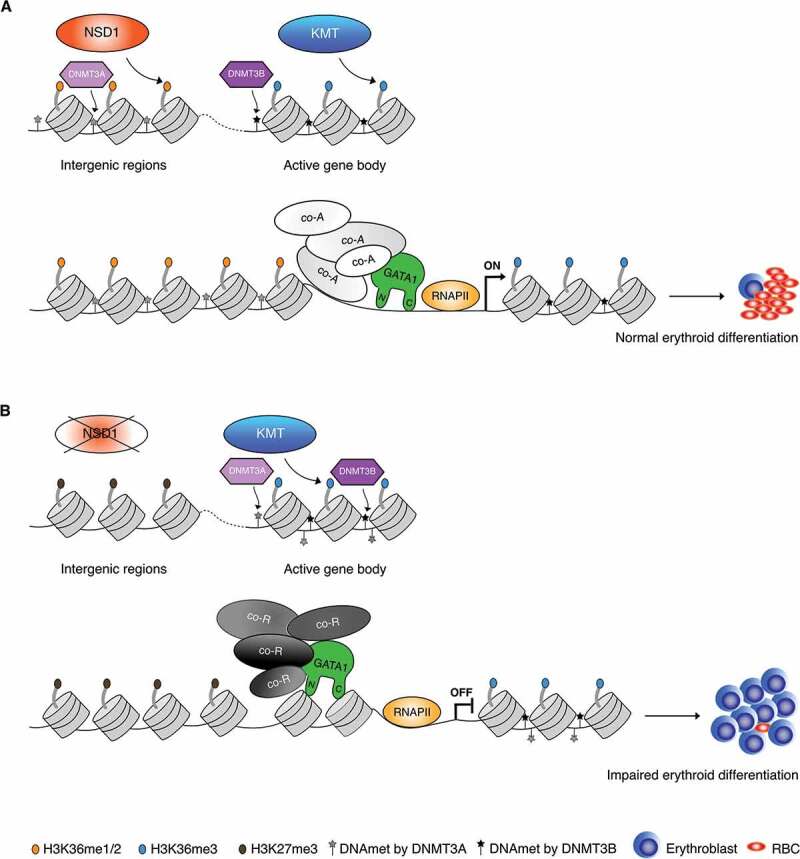
Potential crosstalk between nuclear receptor-binding SET domain protein 1 (NSD1), histone and DNA methylation and GATA1 during erythroid differentiation. (A). NSD1 catalyzes H3K36me1/2 and facilitates H3K36me3 by other histone lysine methyl transferases (KMT) allowing recruitment of DNA methyltransferase 3 alpha (DNMT3A) and DNA methyltransferase 3 beta (DNMT3B) at intergenic and active gene bodies, respectively. GATA1 is able to interact with its co-activators (“co-A”) and together with RNA polymerase II (“RNAPII”) initiates erythroid differentiation and formation of mature red blood cells (RBC). (B). In absence of NSD1, loss of intergenic H3K36me2 marks result in spreading of H3K27me3 at the intergenic regions and dislocation of DNTM3A-facilitated DNA methylation to gene bodies. GATA1 is mostly tethered by co-repressors (“co-R”) and binding to chromatin is reduced resulting in impaired erythroid differentiation and erythroblasts accumulation

Are these findings relevant to understand human cancers? Sequencing the epigenetic landscape of human erythroleukemia and functional *in vitro* and *in vivo* studies strongly proposes that different alterations ultimately converge to impaired GATA1 activity.^9^ The *NSD1* gene is located on the long arm of chromosome 5, a region that is the most frequent target of cytogenetic alteration in erythroleukemia cells.^10^ It is, therefore, possible that reduced NSD1 activity (e.g. by deletion or point mutations) contributes to leukemogenesis by impairing GATA1 transactivation and erythroid differentiation. Most likely aberrant NSD1 activity may contribute to malignant transformation of cells from various tissues by affecting the activity of yet to be identified transcriptional regulators.
